# A hierarchical reinforcement learning method for missile evasion and guidance

**DOI:** 10.1038/s41598-022-21756-6

**Published:** 2022-11-07

**Authors:** Mengda Yan, Rennong Yang, Ying Zhang, Longfei Yue, Dongyuan Hu

**Affiliations:** School of Air Traffic Control and Navigation, Air Force Engineering University, Xian, 710051 China

**Keywords:** Aerospace engineering, Mechanical engineering

## Abstract

This paper proposes an algorithm for missile manoeuvring based on a hierarchical proximal policy optimization (PPO) reinforcement learning algorithm, which enables a missile to guide to a target and evade an interceptor at the same time. Based on the idea of task hierarchy, the agent has a two-layer structure, in which low-level agents control basic actions and are controlled by a high-level agent. The low level has two agents called a guidance agent and an evasion agent, which are trained in simple scenarios and embedded in the high-level agent. The high level has a policy selector agent, which chooses one of the low-level agents to activate at each decision moment. The reward functions for each agent are different, considering the guidance accuracy, flight time, and energy consumption metrics, as well as a field-of-view constraint. Simulation shows that the PPO algorithm without a hierarchical structure cannot complete the task, while the hierarchical PPO algorithm has a 100% success rate on a test dataset. The agent shows good adaptability and strong robustness to the second-order lag of autopilot and measurement noises. Compared with a traditional guidance law, the reinforcement learning guidance law has satisfactory guidance accuracy and significant advantages in average time and average energy consumption.

## Introduction

Target-missile-defender (TMD) engagement has always been a valuable issue, in which a missile struggles to hit the target and a defender aims to intercept the missile. Traditional studies on the TMD problem focus on how the defender can intercept the attack missile^1–4^. With the development of these studies, interception technology has increasingly advanced in recent years. On the other hand, the survivability of attack missiles has been greatly threatened. Therefore, our research focuses on how an attack missile can evade a defender and hit the target. Most contributions have approached this problem using optimal control and differential game theory. Ryoo et al.^5^ studied the evasive manoeuvre strategy of anti-ship missiles against a shipborne close-in weapon system (CIWS) in three-dimensional space. Through the evasive manoeuvre of anti-ship missiles, the CIWS aiming error increases. However, most weapons used in CIWSs are cannons, which are quite different from a homing guidance missile. Yogaswara et al.^6^ studied the evasion strategy of attack missiles against the interceptor missiles of an integrated air defense system. The attack missile needs to avoid interception of the interceptor missiles and eventually guide to the target. A synthesis guidance law is proposed in^6^, which uses an artificial potential field function, time-to-go polynomial guidance law, and logarithmic barrier function to solve the evasion command, impact angle and acceleration constraints, and field-of-view (FOV) constraints. The proposed synthesis guidance law has good performance, however, there are many parameters involved, which make it difficult to choose the optimal value. Qi et al. and Sun et al.^7,8^ derived evasion and pursuit guidance laws for a missile attacking a defended aircraft based on differential game theory. The proposed approach focuses on the miss distance as the outcome of the conflict. Liang et al.^9^ investigated the optimal guidance problem for an interceptor against a ballistic missile with active defence. A class of optimal guidance schemes are proposed based on a linear quadratic differential game method and the numerical solution to the Riccati differential equation. In^9^, the fuel cost, control saturation and chattering phenomenon were considered. Weiss et al.^10^ used a minimum-effort guidance approach to obtain a combined guidance law for the attacker.

Relevantly, the evasive of unmanned aircraft manoeuvring, which is similar to the missile evasion problem, has been studied extensively. Turetsky and Shima^11^ used a matrix game method to study the engagement process of aircraft manoeuvres and homing missiles in the plane. Fonod and Shima^12^ studied aircraft evasive manoeuvring under incomplete information, and an adaptive evasive framework was proposed for the proportional guidance, augmented proportional guidance, and optimal guidance law that the missile may use. Keong et al.^13^ introduced a reinforcement learning method to the issue of avoidance control between aircraft. In^14^, the author used reinforcement learning to train an aircraft agent to continuously avoid multiple surface-to-air missiles, and the agent can control four aircraft simultaneously. This research is valuable, but the aircraft’s decision-making model cannot be directly applied to missiles, because missile guidance not only requires extremely high accuracy but also needs to satisfy several constraints, such as impact angle and FOV constraints. Wang et al.^15^ combined reinforcement learning with a fuzzy method to address a fixed-time pursuit-evasion game. This work shows that artificial intelligence methods, such as, reinforcement learning, can be applied to such pursuit-evasion problems.

As reinforcement learning has recently made remarkable achievements in various fields, its application in missile guidance has gradually become a hotspot. Gaudet, B^16–19^ may be the first to apply reinforcement learning to missile guidance law. The research in^16^ shows that a reinforcement learning guidance law performs better than a proportional guidance method and an enhanced proportional guidance method considering the noise and time delay of the sensors and actuators. In^17^, reinforcement learning combined with meta-learning was applied to the guidance law of exo-atmospheric interceptors. A reinforcement learning algorithm outputs four propulsion instructions for the steering thrusters. The results show that a reinforcement learning guidance law is superior to the traditional zero-effort-miss(ZEM) guidance law in interception rate and energy consumption. Recently, this meta-reinforcement learning method was applied to the hypersonic guidance problem^18,19^. Hong et al. and He et al.^20,21^ studied the comparison between a deep deterministic policy gradient (DDPG)^22^ reinforcement learning guidance law and the traditional proportional guidance law, and experiments verified that a guidance law based on reinforcement learning can be applied to the missile guidance law. In^23^, a double duelling deep Q-network (D3Q)^24^ reinforcement learning algorithm was applied to the mid-course penetration of exo-atmospheric ballistic missiles. However, the evasive manoeuvre of air-to-surface missiles is quite different from the mid-course penetration of exo-atmospheric ballistic missiles.

The purpose of this study is to use reinforcement learning to realize the evasive manoeuvre of an attack missile against an interceptor, and finally guide to the target. To this end, this study first modelled the missile guidance process as a Markov process, with the kinematic relationship between the interceptor, missile, and target as the environment, and the acceleration command as the action of the agent. The hierarchical reinforcement learning method introduces the idea of task decomposition into reinforcement learning, which can reduce the complexity of the problem. Hierarchical reinforcement learning has been adopted in some research studying complex decision problems. Pope et al. and Sun et al.^25,26^ used hierarchical reinforcement learning to address air combat decision-making. In a StarCraft game environment^27^, proposed a hierarchical command and control architecture, consisting of single high-level and multiple low-level reinforcement learning agents operating in a dynamic environment. This hierarchical model enables the low-level unit agents to make individual decisions while taking commands from the high-level commander agent.

The main contributions of this paper can be summarised as follows.

1. A hierarchical reinforcement learning framework for missile evasion guidance is proposed. The entire reinforcement learning framework is divided into two levels. The low level includes two agents, namely, a guidance agent and an evasive agent. The high level is the selection agent, which determines which agent in the low level should be activated in every decision moment.
2. To improve and evaluate the performance of the agents, several metrics are considered, including guidance accuracy, flight time, energy consumption, and an FOV constraint. All agents use the PPO^28^ reinforcement learning algorithm, but each has different observations and rewards.
3. Agents was tested in different scenarios. Experiments show that the hierarchical PPO method is significantly better than the PPO algorithm without a hierarchical structure and a traditional method. Additionally, the agent shows good adaptability and strong robustness to the second-order lag of the autopilot and measurement noises.

The paper is organized as follows. In Section “Problem formulation”, the problem formulation is presented. In Section “Hierarchical reinforcement learning-based guidance law”, the hierarchical reinforcement learning-based guidance law is proposed. In Section “Experiment and result analysis”, experiments and results analysis are presented.

## Problem formulation

### Relative kinematics

**Figure 1:**
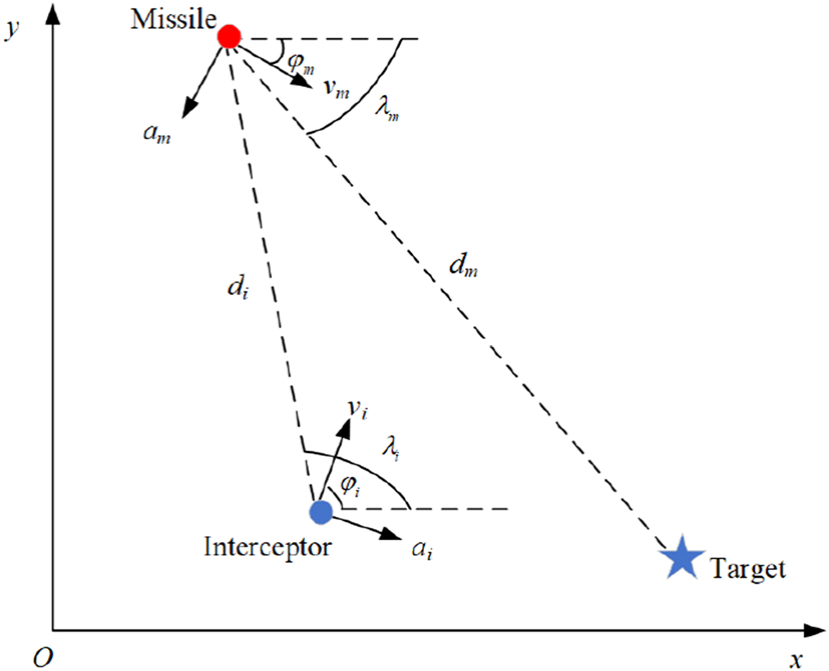
Planar engagement geometry.

The kinematics relationship is shown in Fig. 1. In this paper, we use the term interceptor instead of defender. Therefore, the engagement in this paper can be called the target-missile-interceptor problem. As presented in the geometry, the inertial reference frame is denoted as XOY. The notations $v_{m}$ and ${{v}_{i}}$ are the velocities of the attack missile and interceptor, respectively. The notations ${{a}_{m}}$ and ${{a}_{i}}$ are the lateral accelerations of the attack missile and interceptor, respectively. The notations ${{\varphi }_{m}}$ and ${{\varphi }_{i}}$ denote the flight path angle of the attack missile and interceptor missile, respectively. The notations ${{\lambda }_{m}}$ and ${{\lambda }_{i}}$ denote the line-of-sight(LOS) angle of attack missile to target and interceptor missile to attack missile. The notation of ${{d}_{m}}$ and ${{d}_{i}}$ denote the missile-target relative range and interceptor-missile relative range, respectively.

The equation of motion for both the attack and intercept missiles in the inertial reference is generally given by

1 $$\overset{\centerdot }{\mathop{x}}\,=v\cos \varphi.$$

2 $$\overset{\centerdot }{\mathop{y}}\,=v\sin \varphi.$$

3 $$\begin{aligned}&\overset{\centerdot }{\mathop {\varphi }}\,=\frac{a}{v}. \end{aligned}$$

  The equations describing the interceptor-missile relative motion kinematics can be formulated as

4 $$\overset{\centerdot }{\mathop{{{d}_{i}}}}\,=-{{v}_{i}}\cos ({{\lambda }_{i}}-{{\varphi }_{i}})-{{v}_{m}}\cos (\pi -{{\lambda }_{i}}-{{\varphi }_{m}}).$$

5 $$\begin{aligned}&\overset{\centerdot }{\mathop {{{\lambda }_{i}}}}\,=\frac{{{v}_{i}}\sin ({{\lambda }_{i}}-{{\varphi }_{i}})-{{v}_{m}}\sin (\pi -{{\lambda }_{i}}-{{\varphi }_{m}})}{{{d}_{i}}}. \end{aligned}$$

  Similarly, the equations describing the missile-target relative motion kinematics can be formulated as

6 $$\overset{\centerdot }{\mathop{{{d}_{m}}}}\,=-{{v}_{m}}\cos ({{\lambda }_{m}}-{{\varphi }_{m}}).$$

  

7 $$\begin{aligned}&\overset{\centerdot }{\mathop {{{\lambda }_{m}}}}\,=\frac{-{{v}_{m}}\sin ({{\lambda }_{m}}-{{\varphi }_{m}})}{{{d}_{m}}}. \end{aligned}$$

  In this study, the intercept missile applies typical proportional navigation guidance (PNG) to intercept the attacking missile. The acceleration command for the intercept missile is formulated as

8 $$\begin{aligned} {{a}_{i}}=-N{{V}_{i}}\overset{\centerdot }{\mathop {{{\lambda }_{i}}}}. \end{aligned}$$

where the navigation constant $N=3$.

The lateral acceleration delays generated by the autopilot are also considered. In this study, the missile autopilot controller is modelled as the differential equation of a second-order system as

9 $$\begin{aligned} \overset{\centerdot \centerdot }{\mathop {{{a}_{m}}}}\,=-2\xi {{\omega }_{n}}\overset{\centerdot }{\mathop {{{a}_{m}}}}\,-\omega _{n}^{2}{{a}_{m}}+\omega _{n}^{2}{{a}_{c}}. \end{aligned}$$

where $\xi$ is the damping ratio, ${{\omega }_{n}}$ is the natural frequency, ${{a}_{m}}$ is the derived actuation, and ${{a}_{c}}$ is the acceleration command.

### Engagement scenario

**Figure 2:**
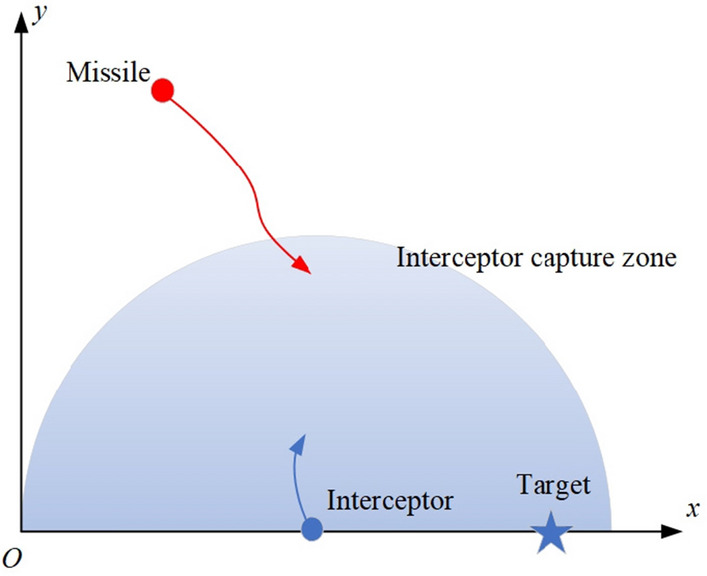
Engagement scenario.

The engagement scenario in this study is shown in Fig. 2. The attack missile performs an evasive manoeuvre first and then guide to the target. To simplify the problem, we assume that the target is stationary, which does not affect the validity of the research, since the simulation focuses on the missile and the interceptor. The intercept missile has a capture zone, which is the maximum launch distance of the interceptor. The interceptor can be launched only when the attacking missile enters the capture zone. The parameters of the engagement scenario are shown in Table 1.

**Table 1:** Parameters of the engagement scenario.

Position of the target	Position of the interceptor launch platform	Range of interceptor capture zone	Flight time of interceptor
(9, 0) km	(5, 0) km	6 km	21 s

**Figure 3:**
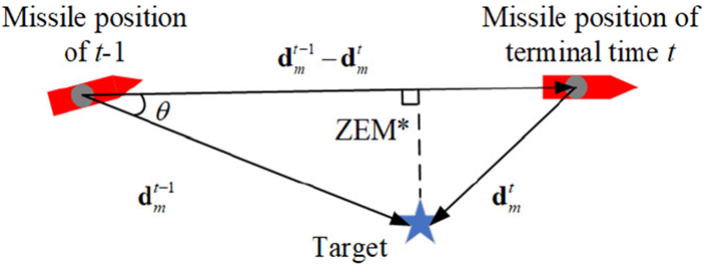
ZEM* Diagram.

The ZEM is an expected miss distance if there is no further manoeuvre from the current location^20,29^. We only consider the ZEM at the terminal state called the ZEM*, which was proposed in^20^. The ZEM* diagram is shown in Fig. 3.

The ZEM* can be calculated by

10 $$\text{ZEM}^{*}=\lvert \mathbf{d}_{m}^{t-1} \rvert\sin \theta.$$

11 $$\theta =\arccos \left( \frac{\left( \mathbf{d}_{m}^{t-1}-\mathbf{d}_{m}^{t} \right)\centerdot \mathbf{d}_{m}^{t-1}}{\lvert \mathbf{d}_{m}^{t-1}-\mathbf{d}_{m}^{t} \rvert\lvert \mathbf{d}_{m}^{t-1} \rvert} \right).$$

It should be noted that the simulation terminal state is defined by the relative distance. The intercepted state and the terminal impact state are defined by judgement terms ${{d}_{i}}\le 10(m)$ and ${{d}_{m}}\le 10(m)$, respectively. The error when using the terminal distance as the judgement condition for simulation termination is acceptable. Once the relative distance is less than 10 metres, it can be considered a hit state. However, due to the limitation of simulation accuracy, the terminal distance is often larger than the ZEM, so it cannot really reflect the guidance accuracy. Therefore, the ZEM* is used to describe the guidance accuracy and used in the reward function, which are described in Section “Agent architecture”.

## Hierarchical reinforcement learning-based guidance law

### Reinforcement learning

**Figure 4:**
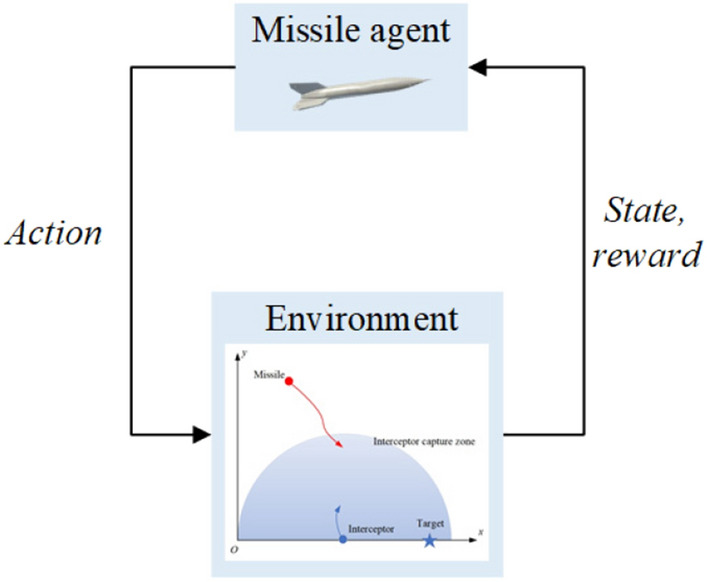
Diagram of reinforcement learning.

Reinforcement learning is a learning process using exploration. The agent learns how to make optimal decisions through continuous interaction with the environment. The agent receives the state of the environment and performs an action based on the state. The environment changes to the next state according to the action and returns a reward or penalty to the agent. The loop continues until the environment reaches the terminal state (success or failure). A simplified diagram of reinforcement learning is shown in Fig. 4.

Reinforcement learning usually models the problem as a Markov decision process (MDP), which comprises a tuple $\left\langle S,A,T,R \right\rangle$. Given a state $s\in S$, selecting an action $a\in A$ will transform the environment to a new state ${s}'\in S$ with transition probability $T(s,a,{s}')\in [0,1]$ and return a reward *R*(*s*, *a*). A stochastic policy $\pi :S\rightarrow A$ is a mapping from states to probabilities of selecting each possible action. The goal is to determine the optimal policy ${{\pi }^{*}}$ that provides the highest expected sum of rewards:

12 $$\begin{aligned} {{\pi }^{*}}=\arg \max {{E}_{\pi }}\left\{ \sum \limits _{t}^{T}{{{\gamma }^{t}}{{r}_{t+1}} \vert {{s}_{0}}=s } \right\} . \end{aligned}$$

where $\gamma \in [0,1]$ is a discount factor. The objective function, called the Q-function, is defined as:

13 $$\begin{aligned} {{Q}_{\pi }}(s,a)={{\text {E}}_{\pi }}\left\{ \sum \limits _{t}^{T}{{{\gamma }^{t}}{{r}_{t+1}}\vert {{s}_{0}}=s ,{{a}_{0}}=a} \right\} . \end{aligned}$$

#### PPO algorithm

The PPO algorithm, which is a state-of-the-art reinforcement learning algorithm, is used to train the agent in this study. The PPO algorithm is an on-policy reinforcement learning algorithm based on an actor-critic (AC) framework. The PPO algorithm uses importance sampling to calculate the ratio of the old policy to the new policy to measure the quality of the new policy, which is shown in Formula (14).

14 $$\begin{aligned} {{p}_{k}}(\theta )=\frac{{{\pi }_{\theta }}({{{\mathbf {u}}}_{k}}\vert {{{\mathbf {o}}}_{k}} )}{{{\pi }_{\theta }}_{old}({{{\mathbf {u}}}_{k}}\vert {{{\mathbf {o}}}_{k}} )}. \end{aligned}$$

  The samples obtained through importance sampling can be reused many times, and the number of samples used, which is defined by the notation $n_{reuse}$ in this paper, is an important hyperparameter in the PPO algorithm. After the PPO algorithm was proposed, several versions have been developed. The most used version is the clip version. The gap between the old policy and the new policy is controlled by a clip function. The objective function is shown in Formula (15).

15 $$\begin{aligned}&J(\theta )={{\text {E}}_{p(\tau )}}\left[ \min \left[ {{p}_{k}}(\theta ),\text {clip}({{p}_{k}}(\theta ),1-\varepsilon ,1+\varepsilon ) \right] A_{{\mathbf {w}}}^{\pi }({{{\mathbf {o}}}_{k}},{{{\mathbf {u}}}_{k}}) \right] . \end{aligned}$$

16 $$\begin{aligned}&A_{{\mathbf {w}}}^{\pi }({{{\mathbf {x}}}_{k}},{{{\mathbf {u}}}_{k}})=\left[ \sum \limits _{l=k}^{T}{{{\gamma }^{l-k}}r({{{\mathbf {o}}}_{l}},{{{\mathbf {u}}}_{l}})} \right] -V_{{\mathbf {w}}}^{\pi }({{{\mathbf {x}}}_{k}}). \end{aligned}$$

Similar to other algorithms with an AC framework, the loss function of the critic network is shown in Formula (17).

17 $$\begin{aligned} L({\mathbf {w}})=\sum \limits _{i=1}^{M}{{{\left( V_{{\mathbf {w}}}^{\pi }({\mathbf {o}}_{k}^{i})-\left[ \sum \limits _{l=k}^{T}{{{\gamma }^{l-k}}r({\mathbf {o}}_{l}^{i},{\mathbf {u}}_{l}^{i})} \right] \right) }^{2}}}. \end{aligned}$$

#### Hierarchical reinforcement learning

A hierarchical structure is used in many traditional action planning methods, such as a hierarchical task network (HTN) planning method^30^. The relationship between high-level tasks and low-level tasks has two types: choice and sequence. The application of this idea to reinforcement learning is called a hierarchical reinforcement learning (RL) method^31,32^. The main advantages of using hierarchical RL are transfer learning (using previously learned skills and subtasks in new tasks), scalability (decomposing large problems into smaller problems, avoiding the problem of dimensionality in high-dimensional state spaces) and generalization (combining smaller subtasks allows generating new skills, superspecialization)^25^.

The PHANG-MAN dog-fight agent^25^ resembles options learning algorithms^33^, and it is closely related to the methods presented in^34^, in which subpolicies are hierarchically structured to perform a new task. In^34^, subpolicies are primitives that are pretrained in similar environments but with different tasks. In^25^, PHANG-MAN has a two-layer policy structure. The high level is the policy selector, which selects one of the low-level policies to activate given the current state of the environment. Similarly, the agent in this study is a two-layer structure, which will be described in detail in the next section.

### Agent architecture

**Figure 5:**
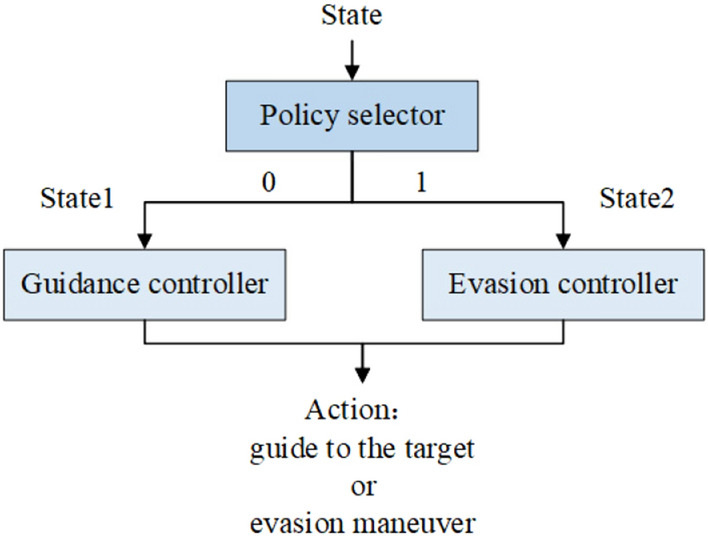
Hierarchical architecture of the agent.

In this study, the missile agent has a two-layer policy structure, as shown in Fig. 5. On the low level, there are two different agents called a guidance controller and an evasion controller, which have been trained in a particular environment state prior to the high-level agent. It should be noted that, the two agents at the low level need different states as inputs. On the high level, there is an agent called a policy selector, whose task is to choose one of the low-level agents to be active at each decision time.

#### Low-level policies

**Figure 6:**
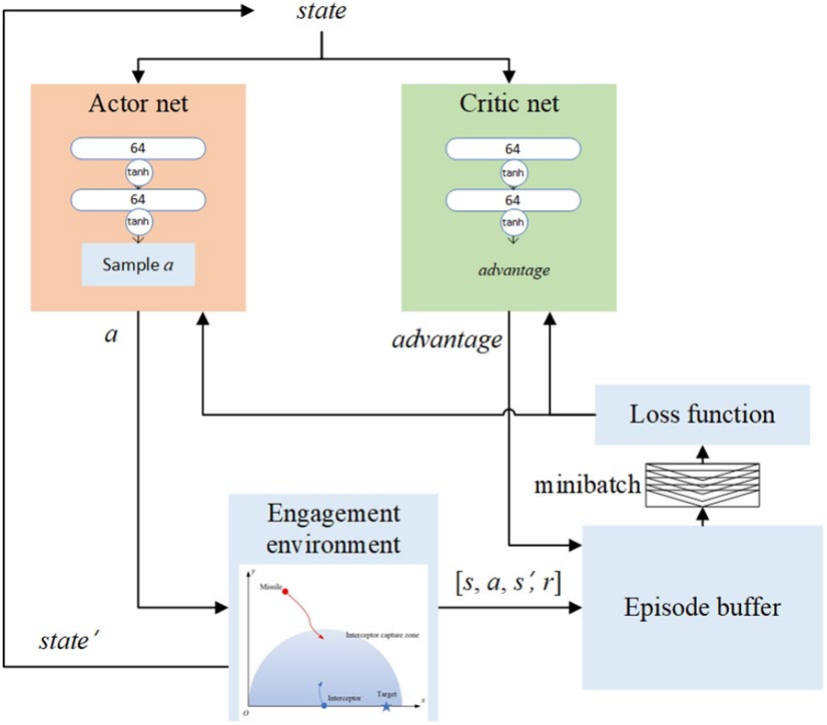
Training structure of the low-level agent.

At the low level, both the guidance agent and the evasive agent are trained using the PPO algorithm with the same neural network structure and action space, but different state space and reward functions. In the PPO algorithm, the actor net and the critic net have the same two-layer network with 64 neurons in each layer. All activation functions are the tanh function. The action space is a continuous interval acceleration command. The state space of the guidance controller agent only considers the relative motion relationship between the missile and the target, that is $stat{{e}_{1}}=\left[ {{d}_{m}},{{\lambda }_{m}},\overset{\centerdot }{\mathop {{{d}_{m}}}}\,,\overset{\centerdot }{\mathop {{{\lambda }_{m}}}}\, \right]$. Similarly, the state space of the evasion controller agent only considers the relative motion relation-ship between the missile and the interceptor, that is $stat{{e}_{2}}=\left[ {{d}_{i}},{{\lambda }_{i}},\overset{\centerdot }{\mathop {{{d}_{i}}}}\,,\overset{\centerdot }{\mathop {{{\lambda }_{i}}}}\, \right]$. The training architecture is shown in Fig. 6.

In the PPO algorithm, parameter sharing between the actor network and critic network is a commonly used tip. Cobbe et al.^35^ studied the performance of sharing parameters in the PPO algorithm and declared that sharing parameters can achieve impressive results in a way, but the hyperparameters must be set reasonably. Since the input and output dimensions of the actor and the critic networks of the low-level agent are the same, it is easy to conduct parameter sharing by combining the loss of the actor and critic networks with appropriate hyperparameters. Maximum entropy reinforcement learning^36^ has achieved impressive results on continuous issues such as walking robots. Adding a policy entropy item to the loss function can enhance the exploration of the agent so it can converge to the optimal state with robustness. The loss function of the low-level agent is formulated as:

18 $$\begin{aligned} \begin{aligned} J(\theta )=-{{\text {E}}_{p(\tau )}}\left[ \min \left[ {{p}_{k}}(\theta ),\text {clip}({{p}_{k}}(\theta ),1-\varepsilon ,1+\varepsilon ) \right] A_{{\mathbf {w}}}^{\pi }({{{\mathbf {o}}}_{k}},{{{\mathbf {u}}}_{k}}) \right] \\ \text { }+\alpha \sum \limits _{i=1}^{M}{{{\left( V_{{\mathbf {w}}}^{\pi }({\mathbf {o}}_{k}^{i})-\left[ \sum \limits _{l=k}^{T}{{{\gamma }^{l-k}}r({\mathbf {o}}_{l}^{i},{\mathbf {u}}_{l}^{i})} \right] \right) }^{2}}} \\ \text { }+\beta \left( -\sum \limits _{x}{p}(x)\log p(x) \right) . \end{aligned} \end{aligned}$$

where the third term is the policy entropy, and $\alpha$ and $\beta$ are hyperparameters.

The reward functions of the guidance controller agent consider guidance efficacy and constraints. The agent needs to trade off guidance accuracy, energy consumption, and flight time to obtain a satisfying guidance performance. Meanwhile, the agent should satisfy the FOV constraint. These reward functions are described as follows.

${{r}_{impact}}$ rewards the agent for mission success when the relative distance between the missile and target satisfies the termination condition. That is

19 $$\begin{aligned} r_{\text{ impact } }= {\left\{ \begin{array}{ll}1, &{} \text{ if } d_{m} \le 10(m) \\ 0, &{} \text{ otherwise } \end{array}\right. } \end{aligned}$$

${{r}_{\text {ZEM}}}$ penalizes the agent for guidance accuracy if the missile hits the target. That is,

20 $$\begin{aligned} r_{ZEM}= {\left\{ \begin{array}{ll}-Z E M^{*}, &{} \text{ if } d_{m} \le 10(m) \\ 0, &{} \text{ otherwise } \end{array}\right. } \end{aligned}$$

${{r}_{out}}$ penalizes the agent for losing the target when the target is out of the FOV. That is,

21 $$\begin{aligned} r_{\text{ out } }= {\left\{ \begin{array}{ll}-1, &{} \text{ if } |\lambda _{m}-\varphi _{m}|>\frac{\eta _{\max }}{2} \\ 0, &{} \text{ otherwise } \end{array}\right. } \end{aligned}$$

where ${{\eta }_{\max }}$ is the maximum FOV of the missile.

${{r}_{a}}$ penalizes the agent for energy consumption. That is,

22 $$\begin{aligned} {{r}_{a}}=-{{\left( \frac{a}{{{a}_{\max }}} \right) }^{2}}. \end{aligned}$$

where *a* and ${{a}_{\max }}$ are the actual acceleration command and the maximum acceleration, respectively.

${{r}_{t}}$ penalizes the agent for flight time. The agent receives a small penalty for every step, which guides the agent to complete the task with as few steps as possible. That is, ${{r}_{t}}=-0.01$.

Note that the first three reward functions are composed of the terminal reward function, which is only given to the agent at the termination state. In Formula (23), ${{r}_{impact}}$ and ${{r}_{out}}$ represent termination conditions. The current simulation terminates once the agent hits the target or the target is out of the seeker’s FOV. If the agent hits the target, ${{r}_{\text {ZEM}}}$ is appended to the reward function at the terminal state as an evaluation metric. The last two reward functions, called shaping reward, are given to the agent at each decision time, to reduce the energy consumption and flight time of the agent.

23 $$\begin{aligned}&{{r}_{\text {terminal}}}={{r}_{impact}}+{{k}_{z}}{{r}_{\text {ZEM}}}+{{r}_{out}}. \end{aligned}$$

24 $$\begin{aligned}&{{r}_{shaping}}={{k}_{a}}{{r}_{a}}+{{k}_{t}}{{r}_{t}}. \end{aligned}$$

where ${{k}_{z}}$, ${{k}_{a}}$ and ${{k}_{t}}$ are hyperparameters.

The reward function for the evasion agent is simpler. The evasion agent will not be rewarded, but will be penalized for being intercepted, losing the target, and energy consumption.

${{r}_{\text {intercpted}}}$ penalizes the agent for being intercepted. That is,

25 $$\begin{aligned} r_{\text{ intercepted }}= {\left\{ \begin{array}{ll}-1, &{} \text{ if } d_{m} \le 10(m) \\ 0, &{} \text{ otherwise } \end{array}\right. } \end{aligned}$$

  The reward function ${{r}_{out}}$ defined in Formula (21) is also applied to the evasion agent. The evasion task is a subtask of the whole task, so the agent is expected to complete the evasion task while not losing the target.

The reward function ${{r}_{a}}$ for the evasion agent is the same as ${{r}_{a}}$ defined in Formula (22), which is the shaping reward.

Similarly, the reward functions of the evasion agent are described as ${{r}_{\text {terminal}}}$ and ${{r}_{shaping}}$. Once the agent is intercepted or loses the target, the current simulation terminates. Meanwhile, the energy consumption should be kept as small as possible during the evasion process.

26 $$\begin{aligned}&{{r}_{\text {terminal}}}={{r}_{\text {intercepted}}}+{{r}_{out}}. \end{aligned}$$

27 $$\begin{aligned}&{{r}_{shaping}}={{k}_{a}}{{r}_{a}}. \end{aligned}$$

  The values of reward hyperparameters of the low-level agents are shown in Table 2.

**Table 2:** Reward hyperparameter values of low-level agent.

Hyperparameters	${{k}_{z}}$	${{k}_{a}}$	${{k}_{t}}$
Value	0.0001	0.001	0.05

#### High-level policy

**Figure 7:**
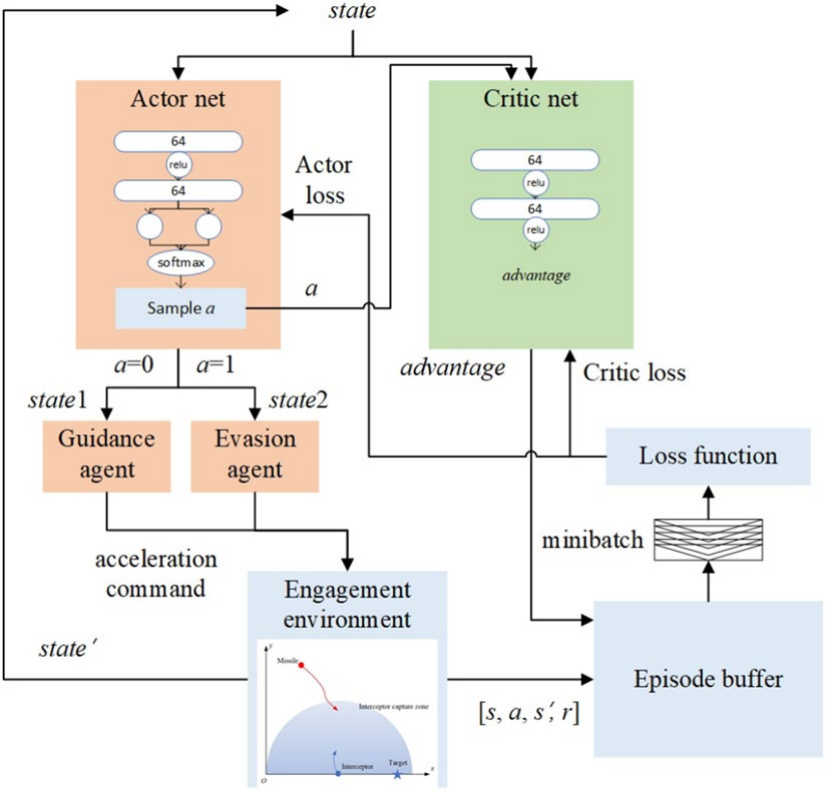
Training structure of the high-level agent.

At the high level, the policy selector agent is also trained using the PPO algorithm. Different from the low-level agent, the action space of the high-level agent is discrete, which can be described as $action\_space=[0,1]$, where 0 and 1 represent guidance and evasion, respectively. Therefore, the activation function of the last layer of the actor net is the softmax function, which can output the probability of sampling each action. The other hidden layers employ RELU activation functions. The state space is the sum of $stat{{e}_{1}}$ and the $stat{{e}_{2}}$, that is, $state=\left[ stat{{e}_{1}},stat{{e}_{2}} \right] =\left[ {{d}_{m}},{{\lambda }_{m}},\overset{\centerdot }{\mathop {{{d}_{m}}}}\,,\overset{\centerdot }{\mathop {{{\lambda }_{m}}}}\,,{{d}_{i}},{{\lambda }_{i}},\overset{\centerdot }{\mathop {{{d}_{i}}}}\,,\overset{\centerdot }{\mathop {{{\lambda }_{i}}}}\, \right]$. The input to the critic net includes the state space plus the action selected at that moment. The training architecture of the high-level agent is shown in Fig. 7. It should be noted that the input and output dimensions of the actor and critic networks are different. The actor network essentially addresses a classification problem, and the classification result needs to be used as the input to the critic network to output a continuous value. It can be said that the optimization goals of the actor and critic networks are inconsistent. According to^35^, the actor and critic networks generally do not share parameters in this case; otherwise, it will have a negative impact on the training process. Therefore, the loss functions of the actor and critic networks are Formulas (15) and (17), respectively. The decision frequency of the agent is 10 Hz. The agent must decide which low-level agent should be activated at each decision moment.

The reward functions ${{r}_{impact}}$, ${{r}_{out}}$ and ${{r}_{\text {intercpted}}}$, which compose the ${{r}_{\text {terminal}}}$, are also applied to the high-level agent.

28 $$\begin{aligned} {{r}_{\text {terminal}}}={{r}_{impact}}+{{r}_{\text {intercepted}}}+{{r}_{out}}. \end{aligned}$$

  To shape the reward, less energy consumption and flight time are also expected. Since these two objects are considered in the low-level agent, the goal of the high-level agent is to select the evasion agent as little as possible while completing the whole task. The ${{r}_{shaping}}$ for the high-level agent is described as

29 $$\begin{aligned} {{r}_{shaping}}=-ka. \end{aligned}$$

where *k* ia s hyperparameter, which is set to 0.001 in the experiment.

## Experiment and result analysis

### Low-level agent training and test

Notably, the training of the low-level agent and the high-level agent are independent of each other. After the low-level agents are trained, they are embedded in the high-level agent. The simulation step is 0.1 s.

#### Guidance agent

A. Training conditions and hyperparameters of the algorithm.

The initialization of the training environment conditions of the guidance agent is shown in Table 3. The relative distance between the missile and the target ${{d}_{m}}$, the LOS angle ${{\lambda }_{m}}$, and the missile heading angle ${{\varphi }_{m}}$ are randomly selected within a certain range in the initial state of each round of training. The speed of the missile ${{v}_{m}}$, and the Cartesian coordinate values of the target are constant values.

**Table 3:** Initial conditions in the training process of the guidance agent.

Parameters	Value
${{d}_{t}}$	$[9,{\text{ }}11]$ (km)
${{\lambda }_{m}}$	$[-\pi /2,\text { }0]$ (rad)
${{\varphi }_{m}}$	$[{{\lambda }_{m}}-\pi /6,\text { }{{\lambda }_{m}}+\pi /6]$ (rad)
${{v}_{m}}$	200 m/s
$x\_\text {target}$	9 (km)
$y\_\text {target}$	0 (km)

The values of the PPO algorithm hyperparameters are shown in Table 4.

**Table 4:** Hyperparameters of the PPO algorithm.

Parameter	Value
$n_{episode}$	500
$n_{step}$	20480
$n_{reuse}$	5
*clip*	0.1
$\alpha$	0.5
$\beta$	0.01
*Gamma*	0.995
*Lamda*	0.95
*lr*	0.0001
*Minibatch*	640

In Table 4, $n_{episode}$ is the maximum number of training episodes, $n_{step}$ is the maximum number of steps of each episode, and *gamma*, *lamda* and *lr* are the hyperparameters in the Adam optimizer^37^.

B. Training result and test.

**Figure 8:**
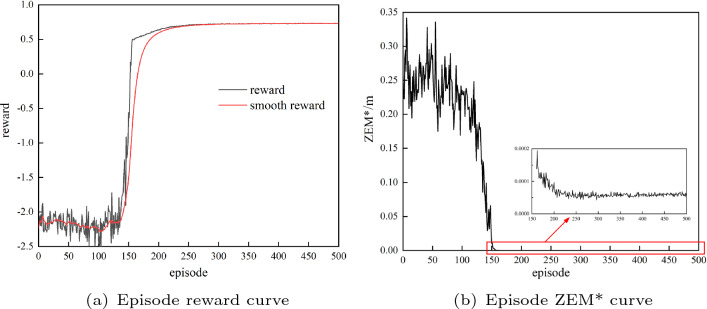
Episode reward and ZEM* of the guidance agent in the training process.

Figure 8a shows the reward curve for the training process of the guidance agent. The training performance was unsatisfactory before 100 episodes, but it quickly improved and converged in a very stable state after 250 episodes. Figure 8b shows the ZEM* curve for the training process. During the training process, the ZEM* dropped from 3000m to approximately 1m, and finally converged to approximately 0.5m, which is a satisfactory result for missile guidance.

**Figure 9:**
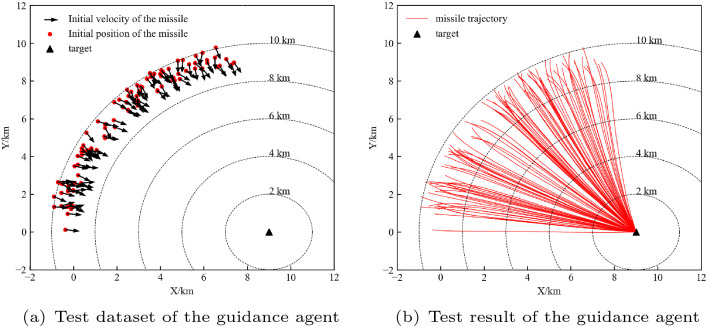
Test dataset and test result of guidance agent.

To test the performance of the guidance agent, we randomly generated 100 initial states to form a test dataset, which is shown in Fig. 9a. Each black arrow is an initial velocity of the missile. Figure 9b shows the performance of the guidance agent on the test dataset. At each initial state, the agent can reach the target with a simple trajectory. This study evaluates the performance of the agent using four metrics: the success rate, ZEM*, average time, and average energy consumption. We define the average energy consumption as:

30 $$\begin{aligned} E=\frac{\sum \limits _{i=1}^{N}{\left( {\sum \limits _{t=1}^{T}{{{a}_{it}}^{2}}}/{T}\; \right) }}{N}. \end{aligned}$$

where N and T are the number of samples and total time of each trajectory, respectively. The data in Table 5 show that the guidance agent has impressive performance.

**Table 5:** Metrics of guidance agent.

Success rate	ZEM*/m	Average time	Average energy consumption/(m s^−2^)^2^
100%	0.31	47.53	44.3

#### Evasion agent

A. Training conditions and hyperparameters of the algorithm.

The initialization of the training environment conditions of the evasion agent is shown in Table 6. The relative distance between the missile and the interceptor ${{d}_{i}}$, the LOS angle of the interceptor ${{\lambda }_{i}}$, and the missile heading angle ${{\varphi }_{m}}$ are randomly selected within a certain range at the initial state. Note that the value of the missile heading angle ${{\varphi }_{m}}$ is still based on the LOS angle of the target ${{\lambda }_{m}}$. The initial velocity vector of the interceptor is pointed toward the missile. The speed of the missile ${{v}_{m}}$, speed of the interceptor ${{v}_{i}}$, and Cartesian coordinate values of the target and interceptor platform are constant values.

**Table 6:** Initial conditions in the training process of the evasion agent.

Parameter	Value
$${{d}_{i}}$$	[4, 6] (km)
${{\lambda }_{i}}$	$[\pi /3, \pi ]$ (rad)
${{\varphi }_{m}}$	$[{{\lambda }_{m}}-\pi /6,\text { }{{\lambda }_{m}}+\pi /6]$ (rad)
${{v}_{m}}$	200 m/s
${{v}_{i}}$	220 m/s
$x\_\text {platform}$	5 (km)
$y\_\text {platform}$	0 (km)
$x\_\text {target}$	9 (km)
$y\_\text {target}$	0 (km)

The hyperparameter values of the PPO algorithm are the same as those in Table 4 shown in Section “Guidance agent”.

B. Training result and test.

**Figure 10:**
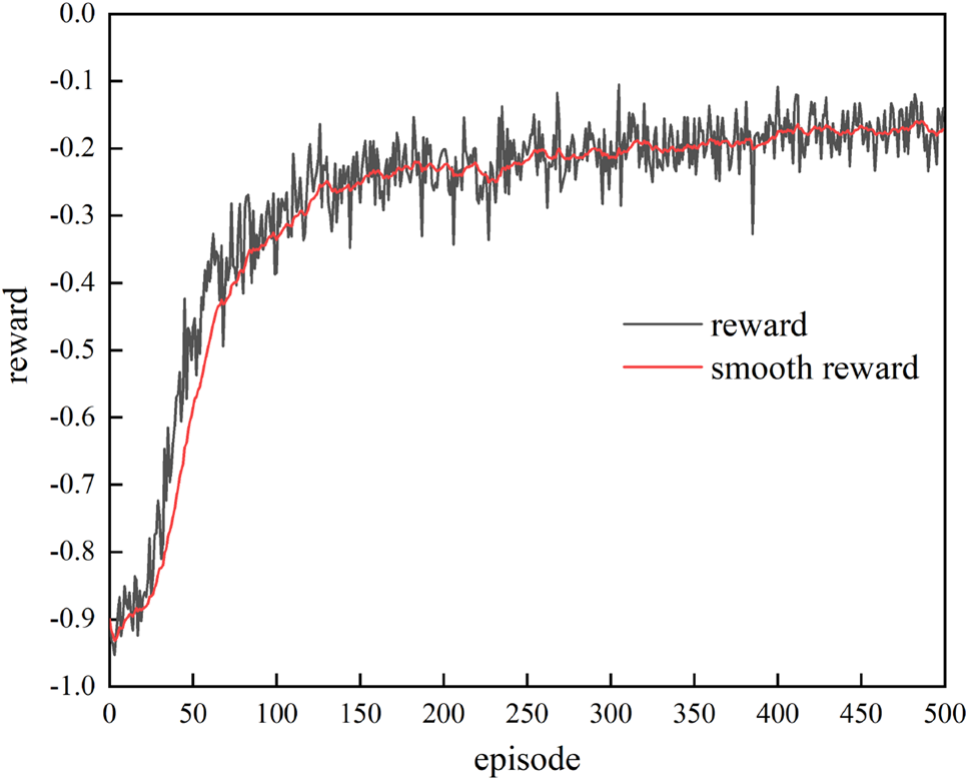
Episode reward in the training of the evasion agent.

The training reward curve of the evasive agent is shown in Fig. 10. The curve is close to convergence after training 200 episodes and approximately obtains the optimal state at the 500th episode.

**Figure 11:**
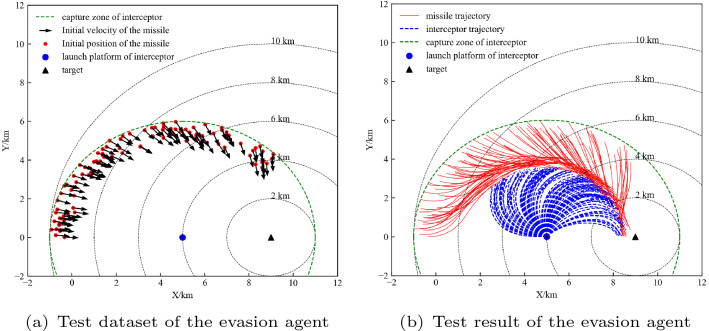
Test dataset and test result of the evasion agent.

To test the performance of the evasive agent, 100 initial states were randomly generated to form a test dataset, which is shown in Fig. 11a. Figure 11b shows the performance of the evasive agent on the test dataset. In each initial state, the agent can quickly make manoeuvres to prevent interception. Only the average energy consumption is used to evaluate the performance of the evasion agent. As shown in Table 7, the average energy consumption of the evasion agent on the test dataset is 225.67(approximately 1.5 g), which is not a very large acceleration command for an evasive manoeuvre.

**Table 7:** Metric analysis of the evasion agent.

Success rate	Average energy consumption/(m s^−2^)^2^
100%	225.67

### High-level agent training and testing

#### Training conditions and hyperparameters of the algorithm

Table 8 shows the initial training conditions for the policy selector at the high level.

**Table 8:** Initial conditions in the training process of the high-level agent.

Parameter	Value
${{d}_{t}}$	$[9,\text { }11]$ (km)
${{\lambda }_{m}}$	$[-\pi /2,\text { }0]$ (rad)
${{\varphi }_{m}}$	$[{{\lambda }_{m}}-\pi /6,\text { }{{\lambda }_{m}}+\pi /6]$ (rad)
${{v}_{m}}$	200 m/s
${{v}_{i}}$	220 m/s
$x\_\text {platform}$	5 (km)
$y\_\text {platform}$	0 (km)
$x\_\text {target}$	9 (km)
$y\_\text {target}$	0 (km)

The values of the hyperparameters of the PPO algorithm, which are shown in Table 9, are slightly different from those of the low-level agent.

**Table 9:** Hyperparameters of PPO algorithm.

Parameter	Value
$n_{episode}$	100
$n_{step}$	10240
$n_{reuse}$	1
*Clip*	0.1
*Gamma*	0.995
*Lamda*	0.95
*lr*	0.0001
*Minibatch*	5120

It was found that the reward converges quickly because the action space of the high-level agent is discrete. However, if the convergence is too fast, the training will be unstable. For instance, the reward curve will quickly rise to a peak and then decrease slowly. For this reason, the hyperparameter $n_{reuse}$ is set to a small value, and *minibatch* is set to a large value so the agent can smoothly converge to the optimal state.

#### Training result and test of the high-level agent

**Figure 12:**
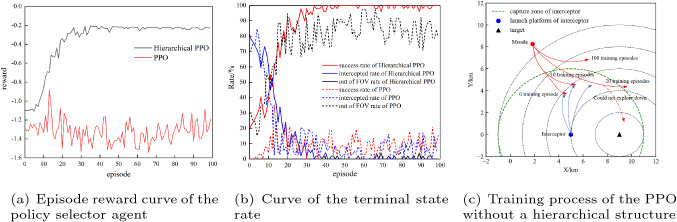
(**a**) shows the episode reward and (**b**) shows the terminal state comparison of hierarchical PPO and PPO. (**c**) shows the training process of the PPO without a hierarchical structure and explains why it fails to complete the task.

In this section, we compare our method with the PPO algorithm without a hierarchical structure. The comparison of training results is shown in Fig. 12. Figure 12a shows the average reward curve for the training process. The proposed hierarchical PPO algorithm has converged when training approximately 40 episodes, while the PPO algorithm without a hierarchical structure never converged. Figure 12b shows the curve of the probability of each termination state in the training process. The success rate of the hierarchical PPO gradually increased during the training process, and the intercepted rate gradually decreased. After 40 training episodes, the success rate reached 100%. In contrast, although the PPO algorithm without a hierarchical structure can also reduce the intercepted rate, the success rate was not improved. The agent converged to the state out of the FOV. Figure 12c shows the training process of the PPO without a hierarchical structure and explains why it fails to complete the task. During the training process, the agent was intercepted at the beginning and then learned to manoeuvre to evade the interceptor. However, the PPO algorithm could not guide the agent to perform a contrary action to explore down in the later training process. Therefore, the agent could not explore the successful terminal state, but converged to a state outside the FOV as soon as possible to reduce the penalty.

**Figure 13:**
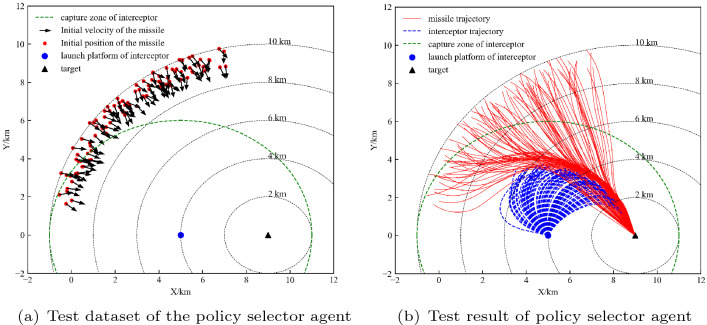
Test dataset and test result of the high-level agent.

Similarly, 100 initial states were randomly generated as a test dataset, which is shown in Fig. 13a. The performance of the agent on the test dataset is shown in Fig.13b, which indicates that the agent can complete the task of evading the interceptor and then reaching the target. Next, we analyse the performance of the agent with the time delay constant of the controller model.

#### Typical scenario analysis

**Figure 14:**
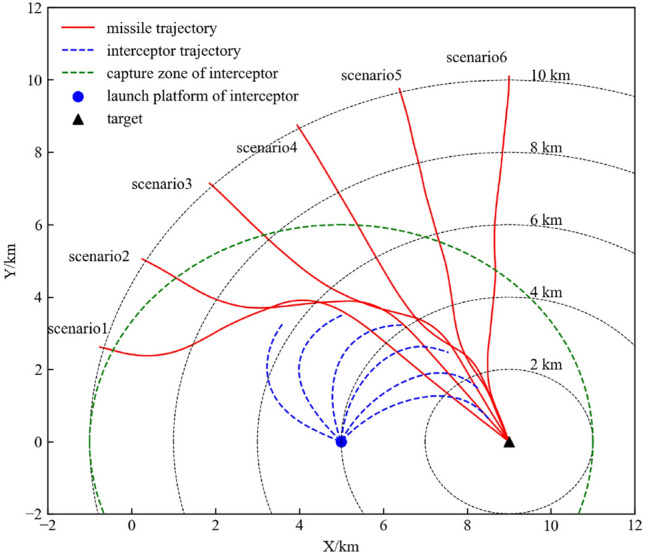
Results of the typical scenarios.

In this section, we select six typical scenarios for detailed analysis. In the six typical scenarios, the initial distance of the missile to the target is 10km, the initial velocity vectors all point toward the target, and the azimuth angles relative to the target are $\frac{\pi }{12}$, $\frac{\pi }{6}$, $\frac{\pi }{4}$, $\frac{\pi }{3}$, $\frac{5\pi }{12}$, and $\frac{\pi }{2}$. The flight trajectories of the agents in the six typical scenarios are shown in Fig. 14.

**Figure 15:**
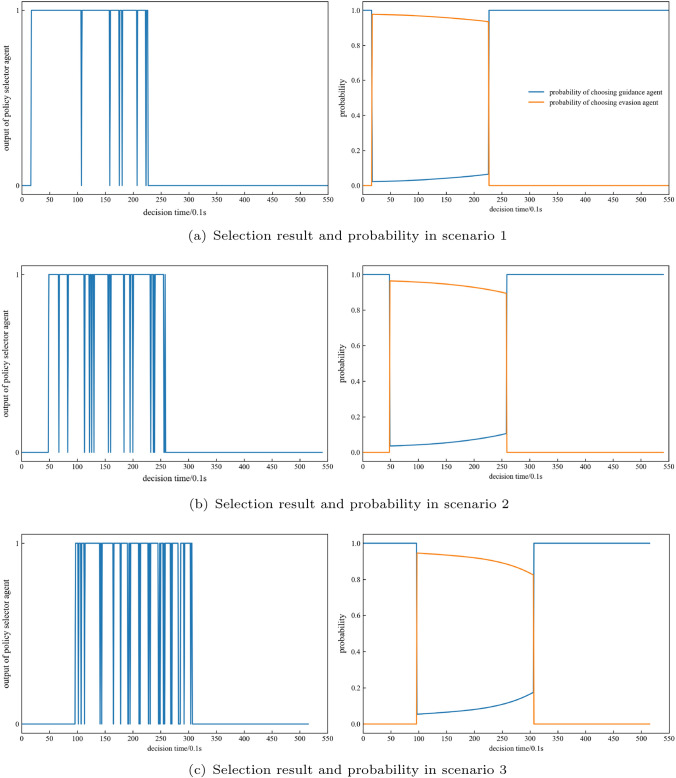

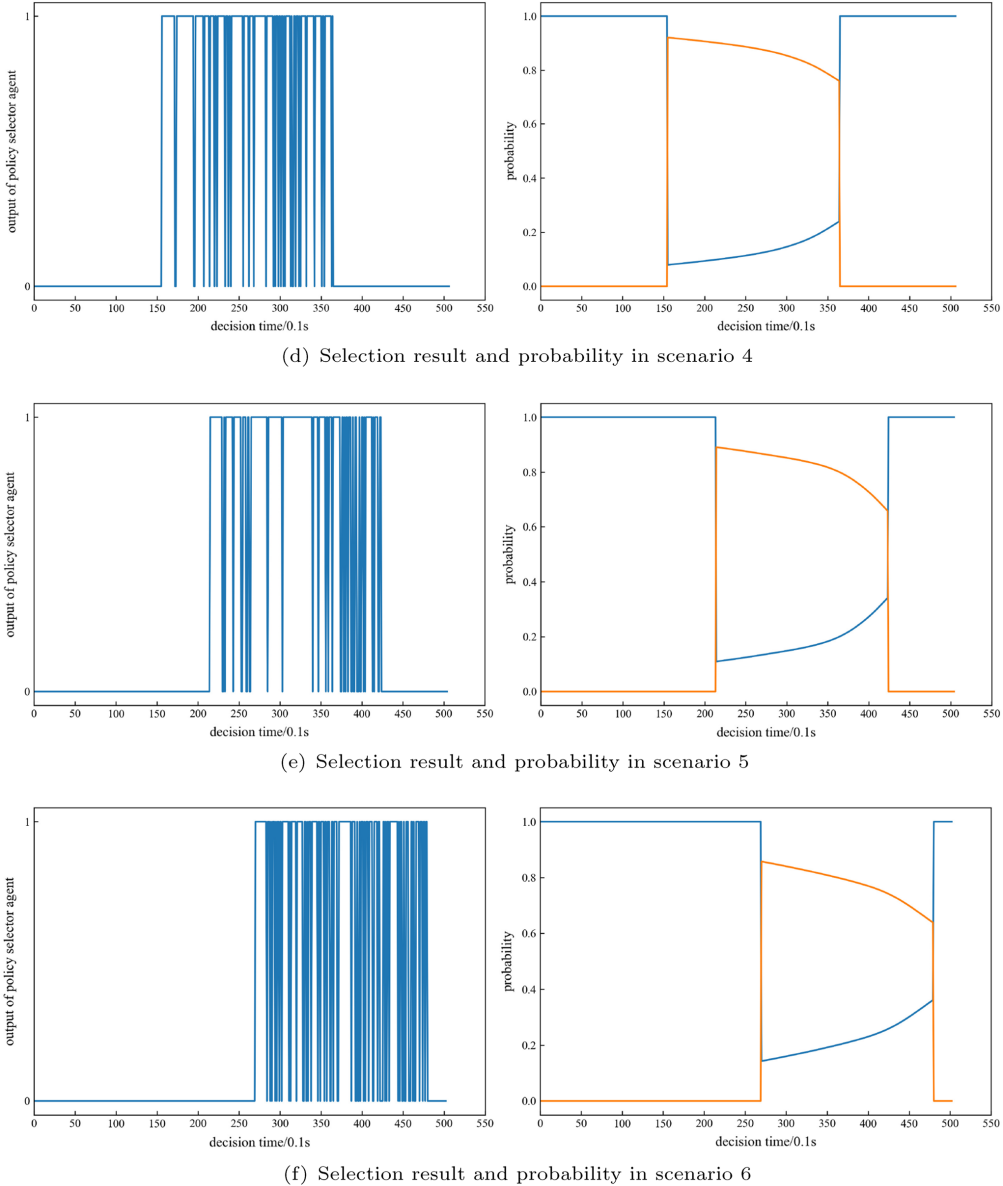
Policy selection analysis for each typical scenario.

First, we analyse the selection of the policy selector agent. The output of the policy selector agent in the six typical scenarios is shown in Fig. 15a–f. The guidance agent is selected when there is no interceptor in the scenario. The figure on the left shows the output of the policy selector agent in the six typical scenarios. 0 indicates the choice of the guided agent, and 1 indicates the choice of the evasive agent. The figure on the right shows the probability of choosing each agent in the six typical scenarios. The yellow curve shows the probability of choosing the evasion agent, and the blue curve shows the probability of choosing the guidance agent. The probability of choosing the evasion agent shows a downwards trend. The policy selector agent generally chooses the evasion agent to control the missile to conduct evasive manoeuvres immediately after the interceptor is launched. However, the probability of choosing the evasion agent may be greatly reduced at the end of the interceptor’s flight time, indicating that the interception capability of the interceptor begins to decline, when the missile can be guided to the target in advance. Representative examples are scenarios 5 and 6. If in a highly threatening scenario, the policy selector agent may choose the evasion agent most of the time; otherwise, it will be intercepted. For instance, in scenario 1, the probability of choosing the evasion agent remained above 0.9 during the whole flight time.

**Figure 16:**
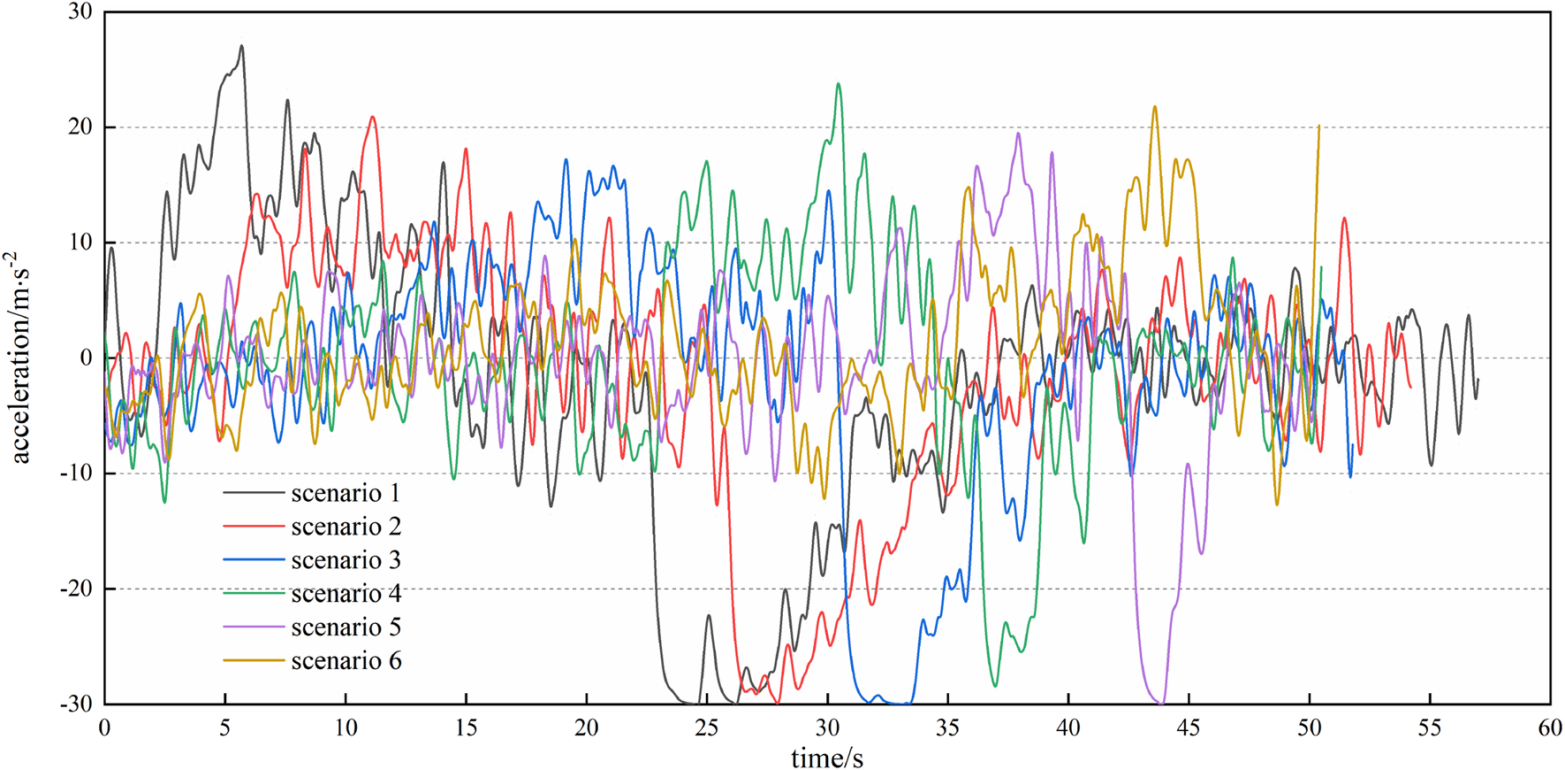
Acceleration analysis for each typical scenario.

Figure 16 shows the acceleration curves of the missile in six typical scenarios. This indicates that the missile rarely uses the acceleration limit value in the evasion process, but the maximum acceleration is often used immediately when the evasion process is completed. The purpose is to complete the task more quickly. The terminal accelerations of the missile are all close to 0 in scenarios 1 to 5, although no terminal acceleration constraints are set in the training algorithm. The reason for scenario 6 having a large terminal acceleration is that the missile is quite close to the target when completing the evasion task. Therefore, a violent manoeuvre must be carried out to guide to the target.

### Robustness experiment

#### Robustness of the autopilot parameters

In this section, we study the robustness of the agent with the autopilot parameters. We add a parameter $\Delta$ to Formula (9) to simulate the uncertainty caused by aerodynamic parameter errors of the missile model^38,39^. Then the autopilot controller can be modelled as

31 $$\begin{aligned} \overset{\centerdot \centerdot }{\mathop {{{a}_{m}}}}\,=-2\xi {{\omega }_{n}}\overset{\centerdot }{\mathop {{{a}_{m}}}}\,-\omega _{n}^{2}{{a}_{m}}+\omega _{n}^{2}{{a}_{c}}+\omega _{n}^{2}\Delta . \end{aligned}$$

**Figure 17:**
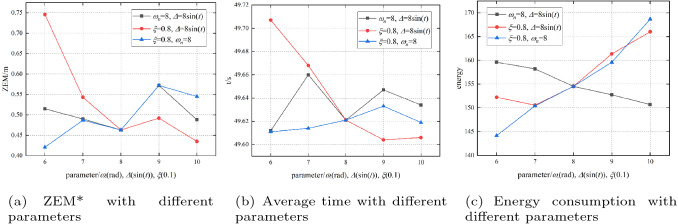
Autopilot parameter analysis.

The default values of parameters $\xi$, ${{\omega }_{n}}$ and $\Delta$ are 0.8, 8 rad/s, and 8sin(t), respectively. The experiment studies the robustness of the agent when $\xi$ changes within the range [0.6, 1], ${{\omega }_{n}}$ changes within the range [8, 10], and $\Delta$ changes from 6sin(t) to 10sin(t). Table 10 shows the experimental results. In Table 10, the success rates are all 100% except for the state when $\xi =1$, which indicates that the agent has good robustness. Figure 17 shows the trend of the metrics when the values of the parameters change.

**Table 10:** Autopilot parameter analysis.

Parameter values			Success rate/%	ZEM*/m	Average time/s	Average energy consumption/(m s^−2^)^2^
$\xi$	${{\omega }_{n}}$	$\Delta$				
0.8	8	8sin(t)	100	0.463	49.621	154.5247
0.6	8	8sin(t)	100	0.515	49.612	159.5974
0.7	8	8sin(t)	100	0.490	49.660	158.2002
0.9	8	8sin(t)	100	0.572	49.647	152.7397
1.0	8	8sin(t)	99	0.488	49.634	150.7082
0.8	6	8sin(t)	100	0.746	49.707	152.2261
0.8	7	8sin(t)	100	0.543	49.668	150.5717
0.8	9	8sin(t)	100	0.492	49.604	161.3431
0.8	10	8sin(t)	100	0.435	49.606	166.0081
0.8	8	9sin(t)	100	0.421	49.611	144.1569
0.8	8	10sin(t)	100	0.487	49.614	150.4246
0.8	8	11sin(t)	100	0.572	49.633	159.5985
0.8	8	12sin(t)	100	0.545	49.619	168.6634

Figure 17a shows that the parameters $\Delta$ and ${{\omega }_{n}}$ can affect the guidance accuracy, while $\xi$ has little effect. The guidance accuracy becomes worse as $\Delta$ increases. In contrast, the guidance accuracy becomes better as ${{\omega }_{n}}$ increases. Figure 17b shows that ${{\omega }_{n}}$ is the main factor that affects the time consumption. Similar to the ZEM, the time consumption becomes better when ${{\omega }_{n}}$ increases, and $\xi$ and $\Delta$ have little effect on the time consumption. Figure 17c shows that the energy consumption is affected by all three parameters. The energy consumption becomes worse as $\Delta$ and ${{\omega }_{n}}$ increase. However, the energy consumption will decrease as $\xi$ increases.

#### Robustness of the information errors

In a realistic engagement scenario, the information is always combined with errors. We study the robustness of the agent considering information errors in this section. The error of the radar information is usually described by the mean squared error (MSE), which is shown as

32 $$\begin{aligned} MSE={\sum \limits _{i=1}^{n}{\bigg |\frac{observation(i)-observatio{{n}_{real}}(i)}{observatio{{n}_{real}}(i)} \bigg |}}\bigg /{n}\;. \end{aligned}$$

**Figure 18:**
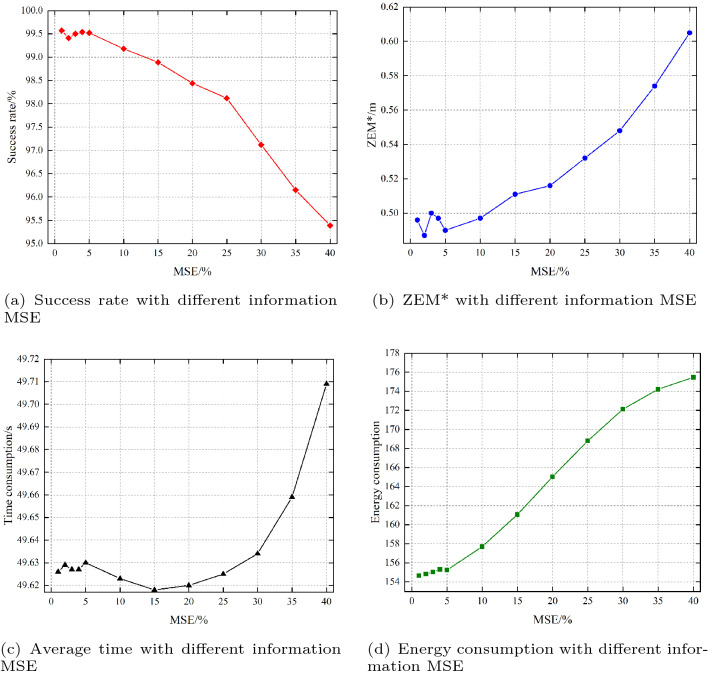
The performance of the agent with different information MSEs.

  Figure 18 shows the experimental results of the performance of the agent with different information MSE. The success rate remains above 99% until the MSE exceeds 10%, and above 98% until the MSE exceeds 25%, which indicates that the agent has impressive robustness to information errors. However, the efficiency of the agent will decrease as the MSE increases, which can be seen in Fig. 18b, c and d.

### Comparison experiment

Finally, this paper compares the proposed hierarchical PPO guidance method with traditional guidance methods based on^6^. A guidance synthesis was designed for this combat scenario in^6^. The acceleration command of the missile consists of three parts: an artificial potential field(APF), polynomial guidance function, and logarithmic barrier function(LBF). The artificial potential field generates evasive acceleration commands. The polynomial guidance function generates guidance acceleration commands considering the impact angle constraints and terminal acceleration constraints. The logarithmic barrier function controls the missile so that it does not exceed the FOV of the missile. Since the impact angle and terminal acceleration constraints are not considered in this study, the polynomial guidance is replaced by a proportional guidance method with N=3. Therefore, the guidance synthesis for comparison in this paper is composed of an artificial potential field, PNG3, and logarithmic barrier function.

The artificial potential field function is formulated as:

33 $$\begin{aligned}&a_{APF}= {\left\{ \begin{array}{ll}F_{\text {repObst}}{{\mathbf {n}}}_{\text {obst}}+F_{\text {repGoal}}{{\mathbf {n}}}_{\text {goal}}, &{} \text{ if } d_{\text {obst}} \le d_{0} \\ 0, &{} \text{ otherwise } \end{array}\right. } \end{aligned}$$

34 $$\begin{aligned}&{{F}_{\text {repObst }}}=\varepsilon \zeta {{d}_{\text {goal }}}{{e}^{-\zeta {{d}_{\text {obst}}}}} \end{aligned}$$

35 $$\begin{aligned}&{{F}_{\text {repGoal }}}=\varepsilon {{e}^{-\zeta {{d}_{\text {obst }}}}} \end{aligned}$$

where ${{{\mathbf {n}}}_{\text {obst}}}$ and ${{{\mathbf {n}}}_{\text {goal}}}$ are the unit vectors of the interceptor pointing to the missile and the missile pointing to the target, respectively; ${{d}_{o}}$ is the distance of the interceptor capture zone; ${{d}_{\text {obst}}}$ and ${{d}_{\text {goal }}}$ are the missile-interceptor relative distance and missile-target relative distance, respectively; $\varepsilon$ and $\zeta$ are hyperparameters.

The PNG3 is formulated as:

36 $$\begin{aligned} {{a}_{PNG}}=-N{{V}_{m}}\overset{\centerdot }{\mathop {{{\lambda }_{m}}}}\ \end{aligned}$$

where N = 3.

The logarithmic barrier function is formulated as:

37 $$\begin{aligned} {{a}_{LBF}}=\left\{ \begin{array}{*{35}{l}} {{a}_{\lim }} &{} \text { if }\lambda \ge b \\ \mu \log (b-|\lambda |) &{} \text { if }b>\lambda \ge \left( b-{{10}^{{}^\circ }} \right) \\ 0 &{} \text { if }\lambda <\left( b-{{10}^{{}^\circ }} \right) \\ \end{array} \right. \end{aligned}$$

where *b* is the maximum FOV angle, $\mu$ is a hyperparameter, and ${{a}_{lim }}$ is the maximum acceleration value.

The acceleration synthesis is:

38 $$\begin{aligned} {{a}_{SYN}}={{a}_{APF}}+{{a}_{PNG}}+{{a}_{LBF}}. \end{aligned}$$

where $\varepsilon$, $\zeta$ and $\mu$ are hyperparameters in guidance synthesis. The values of the hyperparameters are$\mu =5$, $\varepsilon =40$ and $\zeta =3{{e}^{-4}}$, which ensures that the success rate of the guidance synthesis on the test dataset is 100%.

Since the actions of reinforcement learning agents have a certain randomness, we conducted 100 experiments to compare the performance of the reinforcement learning method and the traditional method. Table 11 shows the results of the comparison experiment. It seems that the traditional method is better for guidance accuracy, but the gap is less than 0.02 m and can be ignored. The guidance accuracy of the reinforcement learning method and the traditional method is good enough. Most importantly, the improvements in flight time and energy consumption of reinforcement learning methods are significant.

**Table 11:** Comparison of the hierarchical PPO and Synthesis guidance laws.

Method	Average ZEM*/m	Average time/s	Average energy consumption/(m s^−2^)^2^
Hierarchical PPO	0.441	49.485	153.76
Traditional method	0.428	54.934	161.24

**Figure 19:**
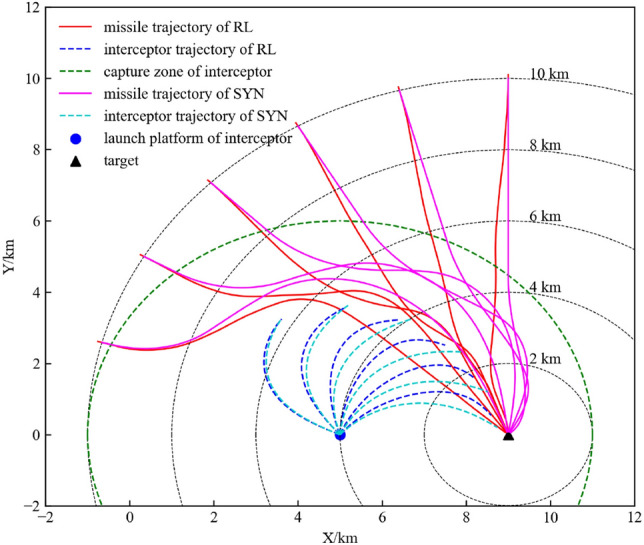
Flightpath comparison of the hierarchical PPO guidance law and synthesis guidance law.

Figure 19 shows the comparison of the missile flight trajectories of the two guidance methods in the six typical scenarios described in Section “High-level agent training and testing”. In the first four scenarios, the missile flight trajectory generated by the guidance law based on hierarchical reinforcement learning is significantly better than the trajectory generated by the synthesis guidance law. The former can concisely complete evasion tasks and quickly guide to the target, while the latter requires a longer flight path. There is an interesting comparison in scenario 5 and scenario 6. The two guidance methods output acceleration commands in opposite directions when evading the interceptor. The synthesis guidance law controls the missile to move away from the interceptor, which is the typical idea. In contrast, the RL-based guidance law first controls the missile to move towards the interceptor for a period of time, and then conducts violent manoeuvres in the opposite direction. Both methods completed the task of evading the interceptor and reaching the target. The RL-based guidance law requires less time. Although it may seem “risky”, this is the advantage of the reinforcement learning guidance law – the agent can find a better solution than experience and knowledge.

## Conclusion

We study the application of reinforcement learning in missile maneuvering. We propose a hierarchical structure and analyse its effectiveness and robustness. Therefore, our research presentation has high professionalism. The conclusions include the following three points:

1. The method based on hierarchical reinforcement learning can complete the task of evading an interceptor and guiding to the target, with a success rate of 100% on a test dataset. The agent performs with great robustness with autopilot parameters and information errors. As the disturbance of the autopilot increases,the metrics of the agent decrease, but it can still almost ensure that the task is completed with a 100% success rate. In an information error experiment, the success rate remained above 99% until the MSE exceeded 10%, and above 98% until the MSE exceeded 25%, which indicates that the agent has impressive robustness to information errors.
2. The PPO algorithm without a hierarchical structure cannot complete the task. Although the agent can learn to not be intercepted, it cannot converge to a state of successfully guiding to the target. The agent finally converges to the state outside the FOV. The method based on hierarchical reinforcement learning can solve this problem.
3. The performance of the proposed reinforcement learning method is better than that of the traditional method, especially considering the average time and average energy consumption metrics. The traditional method has slight advantages in guidance accuracy, while the guidance law based on hierarchical reinforcement learning also has satisfactory guidance accuracy.

In future work, it is necessary to focus on the application of reinforcement learning in the environment of incomplete information and unknown adversaries to improve the robustness and transferability of reinforcement learning agents.

## Data Availability

The datasets and code used and/or analysed during the current study available from the corresponding author on reasonable request.
